# Homing and Detection of Unknown Primary Head-Neck Cancer by Acid-Sensing Nanoparticles

**DOI:** 10.21203/rs.3.rs-8253010/v1

**Published:** 2025-12-10

**Authors:** Jinming Gao, Qiang Feng, Jun Chen, William Hartnett, Sindhu Voorugonda, Brittny Tillman, John Truelson, Larry Myers, Andrew Day, Vijay Basava, Xuechun Wang, Yangyang Zhao, Oreoluwa Onabolu, Natalia Hajnas, Haini Zhang, Gang Huang, Shao-Po Huang, Isaac Chan, Mingyi Chen, Doreen Palsgrove, Samuel Achilefu, Baran Sumer

**Affiliations:** UT Southwestern Medical Center; The University of Texas Southwestern Medical Center; The University of Texas Southwestern Medical Center; The University of Texas Southwestern Medical Center; The University of Texas Southwestern Medical Center; The University of Texas Southwestern Medical Center; The University of Texas Southwestern Medical Center; The University of Texas Southwestern Medical Center; The University of Texas Southwestern Medical Center; The University of Texas Southwestern Medical Center; The University of Texas Southwestern Medical Center; The University of Texas Southwestern Medical Center; The University of Texas Southwestern Medical Center; The University of Texas Southwestern Medical Center; The University of Texas Southwestern Medical Center; University of Texas Southwestern Medical Center; The University of Texas Southwestern Medical Center; The University of Texas Southwestern Medical Center; UT Southwestern Medical Center; The University of Texas Southwestern Medical Center; University of Texas Southwestern Medical Center; University of Texas Southwestern Medical Center

## Abstract

Precise localization of cancer is essential for curative surgery but remains a major clinical challenge when tumors are small or anatomically concealed. While tumor-targeted imaging with nanomaterials has shown promise in preclinical models, mechanistic understanding and clinical translation in cancer patients remain limited. Here, in a prospective Phase 2 clinical trial (NCT05576974), we constructed a single-cell spatial atlas of Pegsitacianine, an acid-sensing nanoparticle with activation pH threshold of 5.3, in human squamous carcinoma of the head and neck. Mechanistically, Pegsitacianine preferentially accumulate in immune-infiltrative severely acidic milieus (iSAM) within tumor stromal regions adjacent to metabolically active cancer cells. Clinically, Pegsitacianine illuminated iSAM regions and achieved fluorescence-guided resection of tumors in 14 of 16 patients with unknown primary cancer, where conventional diagnostic tools failed to locate the tumor. These findings establish the mechanistic link between tumor metabolism, immune infiltration, and nanoparticle delivery, and underscore the clinical value of targeting severe tumor acidosis for cancer detection and therapy.

Surgical resection remains as the foundation of cancer care for many solid tumors, provided the tumor can be accurately located. In head and neck squamous cell carcinoma (HNSCC), tumors can metastasize early to cervical lymph nodes while the primary lesion stays undetectable^1^. This is particularly common in the oropharynx, where a discontinuous basement membrane and complex lymphoepithelial architecture facilitate early lymphatic spread and hinder direct visualization^2–4^. Such cases, known as unknown primary cancer (UPC), may present as cervical nodal metastases or be flagged by circulating tumor DNA analysis^5, 6^, yet still lack an accurate identification of primary tumor. Locating primary occult diseases is critical for allowing curative surgery and minimizing treatment-associated morbidity^7^.

Nanoparticles offer a promising strategy for tumor-targeted imaging and therapy, yet clinical translation has been limited, in part due to unclear and nonspecific targets within human tumors^8–15^. Glycolysis-driven acidification generates distinct microenvironmental features, including focal regions of extracellular acidity^16–22^. To exploit this, we developed Pegsitacianine, an ultra pH-sensitive nanoparticle conjugated with indocyanine green (UPS_5.3_-ICG) that fluoresces exclusively under severe acidic conditions and has demonstrated high tumor specificity in established lesions^23^. In cell culture, where UPS_5.3_ nanoparticles are directly mixed with cancer cells, they revealed a polarized severe acidification phenomenon (pH < 5.3) driven by secretion of lactic acid^22^. However, it remains unknown how acid-sensing nanoparticles recognize severe acidity in human tumors following intravenous administration, and whether they can improve precision surgery of occult diseases when conventional methods are limited.

Herein we initiated a prospective, open-label Phase 2 clinical trial to evaluate Pegsitacianine for intraoperative detection of unknown primary cancers in HNSCC patients (the ILLUMINATE Study, NCT05576974). To investigate the biological mechanism of Pegsitacianine deposition, we generated a single-cell spatial atlas from 13 squamous cell carcinoma patient samples (>1 million cells) collected after systemic administration. Data reveal Pegsitacianine is highly enriched in the severely acidic milieus of tumor stroma with high density of immune infiltrative vasculature. Together, these results provide mechanistic and clinical evidence supporting the use of acid-sensing nanoparticles to target severe tumor acidity for cancer diagnosis and therapy.

## Results

### Acid-sensing nanoparticles illuminate human HNSCC tumors

**Figure 1:**
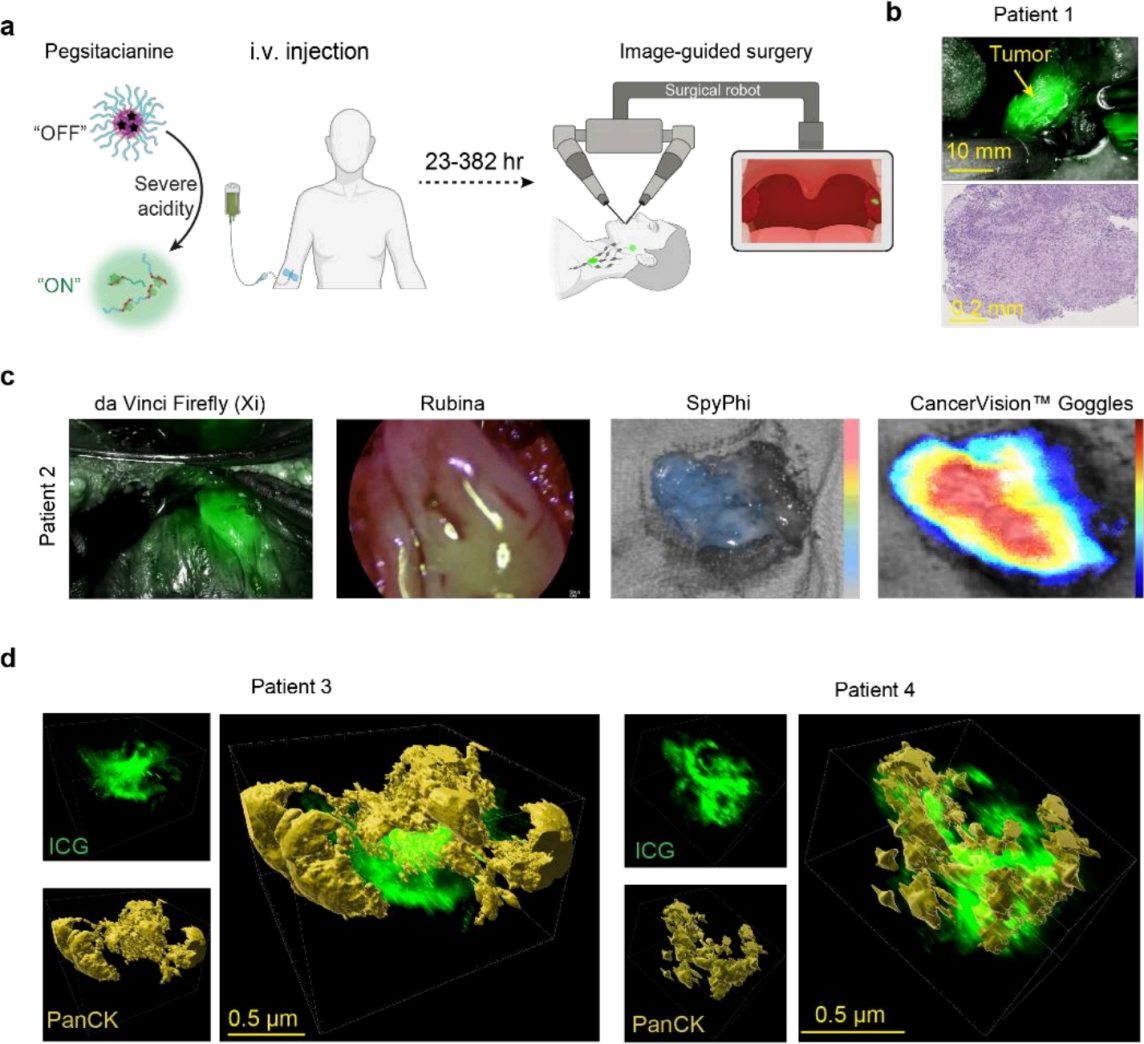
Acidity-targeting nanosensor enables fluorescence-guided detection of human tumors. **a**, Schematic of Pegsitacianine design and clinical workflow. The ultra pH-sensitive nanoparticle remains quenched (“OFF”) at physiological pH but undergoes cooperative disassembly and fluorescence activation (“ON”) under tumor acidity, enabling intraoperative image-guided surgery. **b**, Representative intraoperative fluorescence image from patient 1 showing Pegsitacianine signal (green) localized to a tonsillar tumor, confirmed as squamous cell carcinoma by histopathology. **c**, Pegsitacianine fluorescence detected across four near-infrared platforms, including da Vinci Firefly (Intuitive), Rubina (Karl Storz), SpyPhi (Stryker), and CancerVision^™^ Goggles (prototype), demonstrating cross-platform compatibility. **d,** Threedimensional reconstruction of excised tumor specimens from patients 3 and 4 using tissue clearing and light-sheet microscopy, showing heterogeneous Pegsitacianine accumulation in tumors.

Pegsitacianine is a near-infrared nanosensor designed to undergo sharp off/on fluorescence activation in response to severe acidity (pH < 5.3). The micelle nanoparticle consists of a block copolymer, poly(ethylene glycol)-block-poly(2-(di-n-butylamino)ethyl methacrylate) conjugated with indocyanine green (PEG-*b*-PDBA-ICG). The amphiphilic polymers self-assemble into core-shell micelles with quenched ICG fluorescence at physiological pH, but undergo cooperative disassembly at pH < 5.3 to produce a sharp, binary fluorescence response (Fig. 1a, **Extended Data Fig. 1a**). After disassembly, the positively charged polymers interact with surrounding proteins or cells, creating an integrated fluorescence signal within acidic tumor regions through a “**capture-and-integration” mechanism**^24^. The pH transition was confirmed by titration assays showing a sharp fluorescence change across the transition pH of 5.3 (**Extended Data Fig. 1b**). Importantly, patient plasma samples collected 24 h after intravenous administration retained this switch-like property, showing fluorescence activation at pH 4.5 but not at pH 7.4 (**Extended Data Fig. 1c**), highlighting the stability of the nanosensor in human blood during circulation.

Pegsitacianine was administered intravenously at 1 mg/kg, 23–382 hours before robotic-assisted laryngoscopy biopsy or resection. Pegsitacianine produced robust intraoperative fluorescence signals across patients. In patient 1, tumor region was delineated with strong contrast against adjacent normal tissue, and histopathology confirmed malignant formation (Fig. 1b, **Table S1**). Multi-platform testing showed that Pegsitacianine fluorescence was consistently detected using commercially available da Vinci, Rubina, SpyPhi, cameras and an experimental CancerVision^™^ Goggles (Fig. 1c). These findings demonstrate that acid-sensing nanoparticles effectively accumulate in human tumors.

Spatial heterogeneity in fluorescence intensity and distribution was observed during the trial. To assess this heterogeneity, we systematically biopsied ~300 samples from both fluorescent and non-fluorescent regions in tumor and adjacent tissue and compared fluorescence signal of each sample (**Extended Data Fig. 2a**). Using the presence of identifiable cancer cells by histopathology as the reference, we constructed confusion matrices to quantify the accuracy of fluorescence. Across commercial systems, Pegsitacianine achieved sensitivities ranging from 0.88 to 0.93 and specificities from 0.58 to 0.67. CancerVision^™^ Goggles, which provide continuous fluorescence readouts, yielded an area under the Receiver Operating Characteristic curve of 0.773 across 279 histopathology-confirmed regions (77 cancer cell positive, 202 cancer cell negative) (**Extended Data Fig. 2b**). To further define nanosensor deposition, we applied tissue clearing and light-sheet microscopy to excised tumors. Three-dimensional reconstructions from two representative patients revealed heterogeneous nanosensor accumulation at the cellular level (Fig. 1d). Pan-cytokeratin (PanCK) staining revealed that cancer epithelial regions and nanoparticle imaging signals exhibited complementary spatial patterns within the tumor microenvironment. Together, these results underscore the need for resolving tumor heterogeneity of nanosensor fluorescence at higher resolution.

### Spatial single-cell atlas of Pegsitacianine distribution in HNSCC tumors

**Figure 2:**
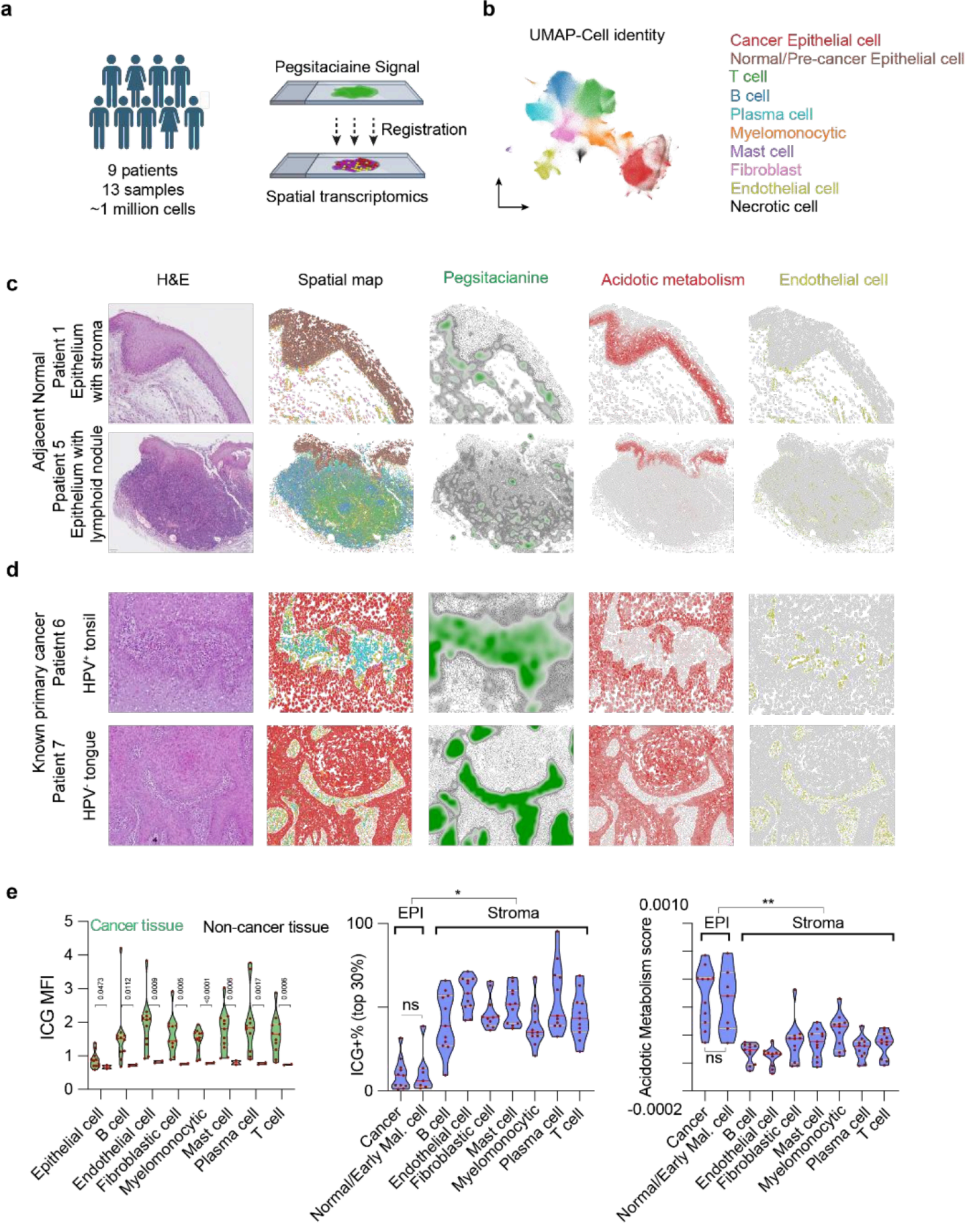
Spatial single-cell atlas of Pegsitacianine distribution in HNSCC tumors. **a,** Schematics of integrating Pegsitacianine fluorescence (ICG signal) with single-cell spatial transcriptomics across 13 tumor sections from 9 patients (~1 million cells). **b,** Uniform Manifold Approximation and Projection (UMAP) of single-cell transcriptomes annotated into 10 major cell populations, including cancer epithelial cells, stromal subsets, and immune cell populations. **c,** Representative fields of adjacent normal tissues showing organized epithelial layers with underlying stroma or lymphoid nodules, where Pegsitacianine activation was sparse. **d,** Invasive HNSCC cancers displayed strong epithelial acidotic metabolism signatures and Pegsitacianine activation in adjacent stromal areas. Acidotic metabolism scores are localized to normal and cancerous epithelial cells, while endothelial cells are restricted to stromal regions. **e,** Quantitative analyses of Pegsitacianine mean fluorescence intensity (MFI), frequency of ICG^+^ cells (top 30%), and acidotic metabolism scores across cell populations. Pegsitacianine activation was highest in stromal cells, whereas acidotic metabolism scores were enriched in epithelial nests. * p<0.05, ** p<0.01

To map tumor distribution of Pegsitacianine at cellular resolution, we combined spatial transcriptomics analysis with fluorescence imaging of tissue sections from freshly resected tumor tissues. Clinical specimens were obtained from 9 HNSCC patients undergoing Pegsitacianine-guided surgery, yielding 13 samples in total (Fig. 2a, **Table S1**). The cohort included 4 patients with human papillomavirus (HPV)-positive head and neck squamous cell carcinoma, several of which were unknown primary cancers (UPCs) that could not be localized by conventional methods but were successfully detected through Pegsitacianine guidance, and 5 patients with HPV-negative disease. For each specimen, Xenium Prime analysis (10x Genomics, 5k human panel) was performed on one section to capture spatial gene expression, while Pegsitacianine fluorescence was acquired and averaged from two immediately adjacent sections (above and below the Xenium section).

Across the dataset, we profiled ~1 million high-quality cells. Unsupervised clustering combined with lineage marker-based annotation identified ten major cell populations representing the tumor microenvironment (Fig. 2b, **Extended Data Fig. 3**), including cancer epithelial cells, normal or pre-cancer epithelial cells, fibroblasts, endothelial cells, T cells, B cells, plasma cells, myelomonocytic cells, mast cells, and necrotic cells. Tumor epithelial clusters expressed canonical squamous carcinoma markers such as *CLDN1, LAMB3, TP63, CDKN2A, EPCAM*, and *SERPINB2*, validating their malignant identity, while stromal and immune subsets displayed expected transcriptional programs. Spatial distributions of major cell types were further confirmed by histopathologic review of matched histological sections. Consistent with known pathology, cancer and normal epithelial cells formed compact nests with limited vascularization and sparse immune infiltration, whereas immune and endothelial cells were concentrated in surrounding stromal regions.

To integrate fluorescence signal with transcriptomics, the two flanking slides with ICG imaging data were co-registered to the central Xenium slide using the Warpy workflow (**Extended Data Fig. 4**). The ICG signal was imputed onto the center Xenium slide by averaging and smoothing values from the adjacent slides for display. Similar to that observed in patient imaging and 3D cleared tissues, Pegsitacianine fluorescence exhibited pronounced spatial and intra-sample heterogeneity (**Extended Data Fig. 5**). The fluorescence map delineated regional acidic zones within tumors, rather than strictly cell-confined patterns, indicating fluorescent pixels could fall inside or outside cellular boundaries. To enable integrated analysis with single-cell transcriptomics, we used Mask2SC workflow^25^ to assign ICG mean fluorescence intensity (MFI) values to individual cells as carriers of the local signal. While this approach discards extracellular pixels, it allows downstream analysis within the framework of single-cell datasets. In this way, the combined transcriptomic and fluorescence data provide the first human tumor-based single-cell spatial atlas of nanoparticle distribution.

### Homing of Pegsitacianine in stromal areas surrounding metabolically active cancer nests

We analyzed representative regions from normal head and neck tissues adjacent to tumors and from invasive cancer specimens. Overall, normal tissues displayed only sparse, low-intensity fluorescence compared to invasive cancers. Two representative types of normal tissues were selected: epithelium with connective tissue stroma and epithelium with lymphoid nodules (Fig. 2c). Sparse ICG signals were observed in stromal regions. To further assess metabolic activity, we calculated an acidotic metabolism score using genes involved in glycolysis (HK2, ENO2, PGK1, PKM, ALDOA, HK1, PFKFB3, LDHA), Pyruvate oxidation/TCA cycle/electron transport chain (PDHA1, PDHB, DLD, CYCS, ATP5F1A, ATP5F1C, SDHA, SDHB, NDUFAB1), lactic acid and CO_2_ transport (SLC16A1, CA9, CA12), and visualized its spatial expression. High scores are localized primarily in the basal layer of the epithelium, consistent with the proliferative and metabolically active state of basal cells. Endothelial cells were largely excluded from epithelial regions, consistent with the avascular nature of epithelium, and overlapped with acidity signal. These data highlight that intravenously injected UPS nanoparticles show no major accumulation in normal tissues, with fluorescence largely restricted to the inside of vessels.

Compared with tumor-adjacent normal tissues, invasive cancers exhibited markedly higher ICG signals (Fig. 2d). Similar to normal tissue, Pegsitacianine activation is localized predominantly in tumor stromal regions enriched with endothelial cells, whereas acidotic metabolism genes were concentrated in epithelial nests rather than stroma. Histological and spatial transcriptomic analyses revealed a complementary pattern: metabolic activity is enriched within cancer epithelial compartment, whereas Pegsitacianine accumulation in surrounding stromal areas.

Quantitative analysis of the integrated dataset confirmed this spatial segregation (Fig. 2e). Across all samples, Pegsitacianine mean fluorescence intensity (MFI) was significantly higher in cells from invasive cancer samples compared with adjacent non-cancerous tissues. Within cancer samples, stromal cells exhibited much stronger fluorescence than epithelial cells, whereas acidotic metabolism scores were enriched in epithelial nests but not in other compartments. This pattern was conserved across both HPV-positive (**Extended Data Fig. 6**) and HPV-negative (**Extended Data Fig. 7**) tumors. Although preclinical studies have highlighted macrophages as key mediators of nanoparticle transport in animal models^26^, our data indicate that acidity-associated fluorescence in human tumors does not map to a single immune cell lineage, including myelomonocytic cells. Together, these findings support a model in which acidity produced by metabolically active epithelial nests diffuse into surrounding stroma, where it establishes severely acidic milieu that serve as primary sites for nanoparticle accumulation.

### Severely acidic milieu associates with immune infiltrating vasculature

**Figure 3:**
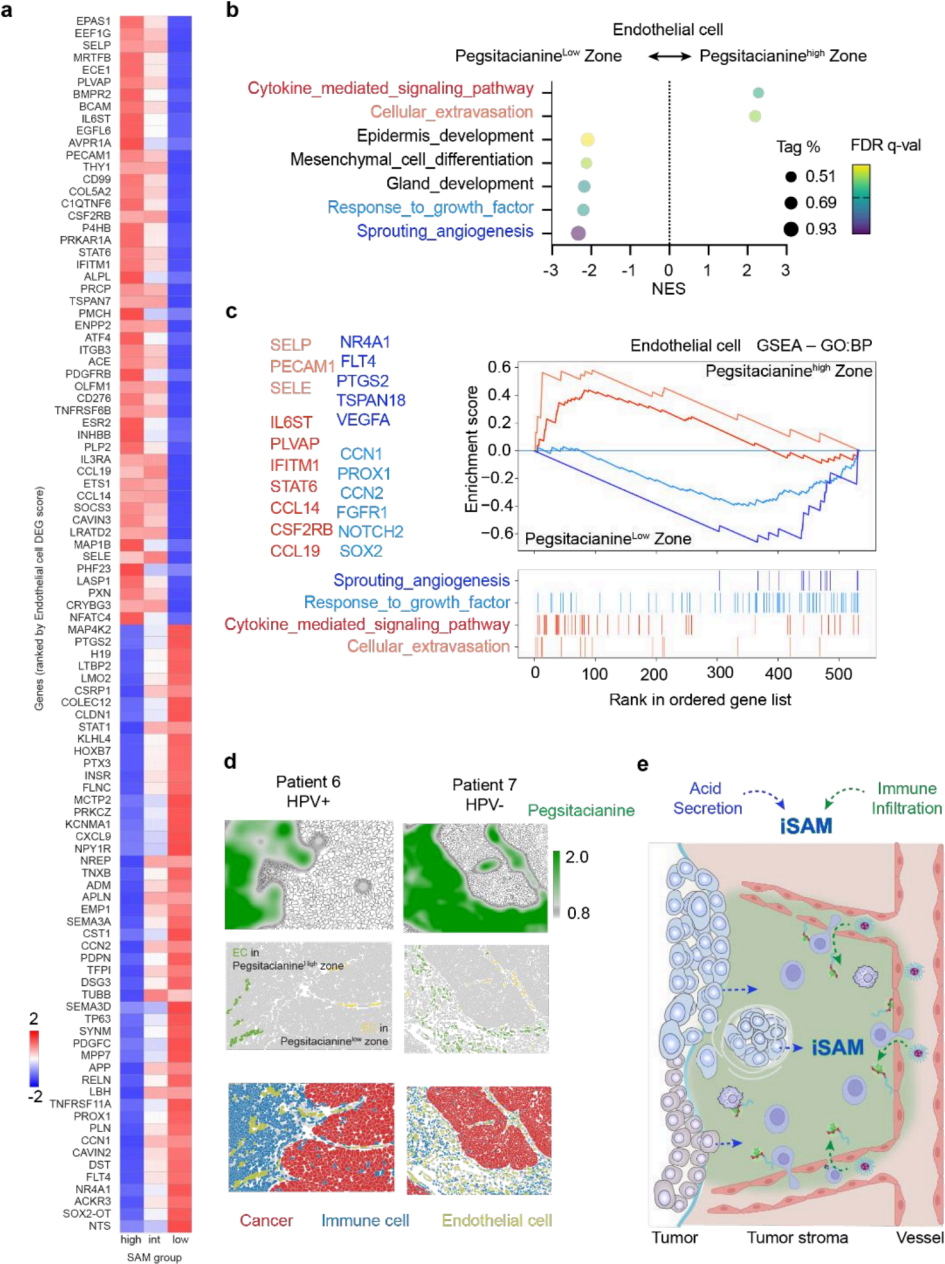
Severe acidity milieu (SAM) associates with immune-infiltrating vasculature. **a**, Heatmap of differentially expressed genes in endothelial cells from SAM–high (Pegsitacianine^high^) versus SAM–low (Pegsitacianine^low^) regions. **b**, Gene set enrichment analysis (GSEA) of endothelial cells shows positive enrichment of cytokine-mediated signaling and cellular extravasation in SAM–high regions, and negative enrichment of angiogenesis- and growth-related pathways. **c**, GSEA plots highlighting upregulated inflammatory pathways (e.g., cytokine signaling, leukocyte adhesion) and downregulated sprouting angiogenesis in SAM–high endothelial cells. **d**, Spatial mapping of SAM–high and SAM–low zones in HPV-positive and HPV-negative tumors, showing endothelial distribution relative to cancer and immune cells. **e**, Schematic model: acidity produced by tumor epithelial nests diffuses into the stroma, where inflamed endothelial cells, recruit immune cells, and promote nanosensor activation at vascular entry points.

To characterize the endothelial compartments governing the entry of nanoparticles, we classified stromal regions into Pegsitacianine^high^ and Pegsitacianine^low^ zones based on ICG intensity across non-epithelial cells within each sample. Endothelial cells in these regions were then designated as Pegsitacianine^high^ or Pegsitacianine^low^ endothelial cells, respectively. Differential gene expression analysis revealed that endothelial cells in Pegsitacianine^high^ zones upregulated cytokine response genes (*SELP, IL6ST, STAT6*) and leukocyte adhesion/extravasation markers (*PECAM1, PLVAP, SELE*), while downregulating angiogenesis- and growth-associated genes (*VEGFA, FLT4, FGFR1, CCN2, SOX2*) (Fig. 3a). Gene set enrichment analysis further supported this phenotype, showing positive enrichment of cytokine-mediated signaling and cellular extravasation, but negative enrichment of sprouting angiogenesis and vascular growth pathways (Fig. 3b–c). Field-of-view images confirmed these differences: endothelial cells in Pegsitacianine^high^ zones were surrounded by dense immune infiltrates, whereas endothelial cells in Pegsitacianine^low^ zones were more isolated and extended into tumor nests (Fig. 3d).

Together, these findings indicate that Pegsitacianine accumulation in tumors relies on infiltrative endothelium that facilitates immune cell/nanoparticle import as well as acid export. They point to the existence of an immune-infiltrative severely acidic milieu (iSAM) within the tumor stroma, where immune cell trafficking, acid buildup, and nanosensor activation converge at the tumor–stromal interface (Fig. 3e).

### Pegsitaciaine identified unknown primary cancer and altered clinical outcomes

**Figure 4:**
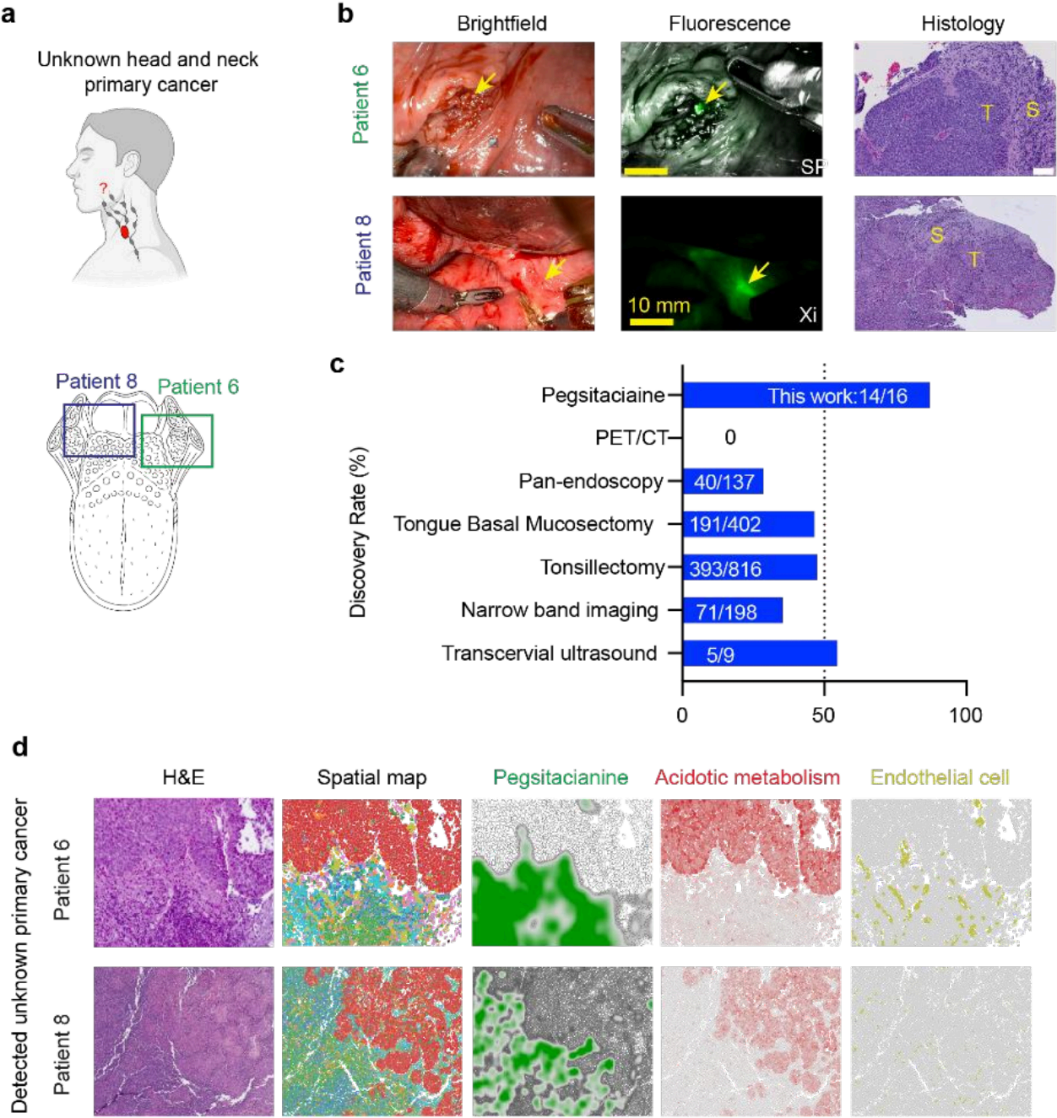
Pegsitacianine imaging identifies unknown primary cancer in the head and neck. **a,** Schematic depicting the clinical challenge of unknown primary cancer (UPC), in which patients present with nodal metastases or other signs of malignancy without a detectable primary tumor. The lower panel shows the location of UPCs in two representative patients. **b,** Intraoperative brightfield, fluorescence, and hematoxylin and eosin (H&E) images demonstrating Pegsitacianine-guided detection of UPCs in two patients, as confirmed by histopathology. **c,** Bar graph comparing the detection rate of Pegsitacianine (14/16 in this study) with reported rates for positron emission tomography/computed tomography (PET/CT), transcervical ultrasound, narrow band imaging, and operative laryngoscopy. **d,** Spatial transcriptomic maps of two representative UPC samples showing Pegsitacianine accumulation in stromal regions adjacent to acidotic metabolism–rich epithelial nests, with colocalized vasculature and immune infiltration signatures.

As part of the Phase II ILLUMINATE trial (NCT05576974), we enrolled 16 HPV-related, p16-positive patients with unknown primary cancers (UPCs, Fig 4a, **Table S1**). All subjects presented metastatic squamous cell carcinoma to cervical lymph nodes, and their HPV status was confirmed by p16 immunohistochemistry and/or HPV DNA in situ hybridization. As part of the diagnostic work-up, each subject underwent head and neck physical examination including fiberoptic laryngoscopy, computed tomography, and ^18^F-fluorodeoxyglucose positron emission tomography (FDG-PET). In 15 of the 16 subjects, no primary tumor could be localized by these standard procedures, while one subject (patient 6) had a right tonsillar carcinoma without additional identifiable disease.

Pegsitacianine-guided fluorescence identified occult primary tumors in 14 of 16 cases. Representative intraoperative images and videos show clear detection of UPC from the surrounding normal tissue (Fig. 4b, **Extended Data Fig. 8**, **Supplementary Videos 1–5**). In patient 6, fluorescence not only confirmed the right tonsil tumor but also revealed an additional undetected contralateral tonsillar lesion that was subsequently resected. All fluorescence-positive lesions were confirmed malignant on histopathology. The average tumor diameter was 7.2 mm (range 2–15 mm, **Table S1**). Compared to historical reports of transoral ultrasound, narrow band imaging, or operative laryngoscopy alone, Pegsitacianine achieved the highest reported UPC detection rate (Fig. 4c, **Extended Data Fig. 9**).

Importantly, intraoperative localization altered clinical management. In eight patients, fluorescence guidance enabled more conservative resections with reduced tissue loss. Eleven patients received lower radiation doses and volumes; eleven subjects also avoided adjuvant chemotherapy, with three required no further therapy, including radiation. Mechanistically, spatial profiling of representative UPC specimens revealed that Pegsitacianine accumulated in stromal regions adjacent to metabolically active epithelial nests, co-localizing with endothelial cells and immune infiltrates (Fig. 4d, **Extended Data Fig. 6**), similar to established HNSCC tumors. These findings underscore iSAM as a viable and translatable target that offers clinical value in cases where other diagnostic tools have shown limitations.

## Discussion

In HPV-associated UPC of the head and neck, accurate resection of the occult tumors enables curative therapy; however, this is often challenging. Traditional transoral surgical excision without image guidance involves trial resection of at-risk tonsillar tissue, but tumors are frequently discovered post hoc, and large areas of the oropharynx, typically the ipsilateral tonsil and often the bilateral lingual tonsils, are routinely removed, increasing morbidity. Following surgery, patients may require adjuvant radiation or chemotherapy when the primary tumor cannot be successfully identified and fully resected, which carries substantial long-term toxicities^27, 28^. In this study, Pegsitacianine-guided imaging identified tumors as small as 2 mm, enabling precise, tissue-sparing excisions and avoiding removal of large portions of oropharyngeal structures, high-dose radiation, and extensive adjuvant chemotherapy. These findings demonstrate that Pegsitacianine imaging through intraoperative fluorescence introduces an orthogonal information stream to conventional imaging and clinical evaluation, offering a clinically valuable strategy for precise surgical resection of tumors, especially when other methods fail.

Tumor targeting by nanoparticles holds transformative potential for cancer treatment. Nanomaterials have been widely investigated to achieve targeted drug delivery or imaging in tumors through the leaky vessels described by the enhanced permeability and retention (EPR) effect^29, 30^, with research spanning over three decades^29, 31, 32^. Numerous attempts to exploit the EPR effect for tumor targeting have fallen short in clinical settings, raising fundamental questions about how nanoparticles accumulate inside tumors^10^. Preclinical studies have shown that passive leakage accounts for only a small fraction of tumor accumulation, leading to the active transport and retention (ATR) hypothesis, which posits that nanoparticle delivery (for example, gold nanoparticles) is primarily driven by energy-dependent processes such as endothelial transcytosis^8, 15, 33^. Yet direct evidence supporting these mechanisms in human tumors remains scarce, highlighting a critical gap in our understanding of nanoparticle behavior in patients. In this study, we identified the accumulation zones of Pegsitacianine as iSAM and propose that tumor acidity triggers micelle disassembly and local retention, while infiltrative endothelium permits both immune cell and nanoparticle entry (as well as acid export). These findings align with the emerging ATR framework but suggest a distinct mechanism: rather than transcytosis through endothelial cells, material exchange may occur alongside immune infiltration, where acidity and cellular trafficking converge to drive nanoparticle accumulation in solid tumors.

In summary, this study presents a human tumor-based single-cell spatial atlas of Pegsitacianine accumulation and identifies iSAM as the metabolic target for tumor illumination. Pegsitacianine-guided imaging provides an intraoperative strategy for real-time detection and resection of occult malignancies as small as 2 mm. As liquid biopsy technologies continue to mature for early cancer detection, pairing them with imaging approaches such as Pegsitacianine offers an integrated framework for early detection, anatomical localization, and treatment.

## Methods

### Pegsitacianine

Pegsitacianine was manufactured under Good Manufacturing Practice (GMP) conditions by OncoNano Medicine, Inc. (Southlake, TX, USA) in compliance with the U.S. Food and Drug Administration (FDA) regulations. The final product was formulated, tested, and released for clinical use following standard operating procedures and quality control criteria. The drug product was supplied as a sterile aqueous formulation in vials containing 10 mg Pegsitacianine polymer in 10% (w/v) trehalose solution. The material was diluted in sterile saline for intravenous infusion at a final dose of 1 mg/kg. All clinical doses were prepared and administered by qualified pharmacy staff at the University of Texas Southwestern Medical Center.

### Other materials

Spectral DAPI (Cat#: FP1490) was purchased from Akoya Biosciences. Xenium Prime 5K Human Pan Tissue & Pathways Panel (Cat#: 1000671) was from 10x Genomics. ProLong^™^ Diamond Antifade Mountant (Cat#: P36961) was from Invitrogen. Alexa Fluor^®^ 647 Anti human pan-cytokeratin was from Abcam (ab309978). All other routine laboratory reagents and chemicals were obtained from Fisher Scientific or Sigma-Aldrich.

### Study design

The ILLUMINATE study (NCT05576974) is a Phase2, prospective, single-arm, open-label clinical trial designed to evaluate the feasibility of Pegsitacianine-guided fluorescence imaging for intraoperative detection of UPC in HNSCC patients. Eligible participants were adults (aged ≥18 years) with head and neck squamous cell carcinoma undergoing surgical excision. HPV status was confirmed by p16 immunohistochemistry and/or HPV DNA in situ hybridization. Participants were required to have a performance status suitable for surgical intervention and no contraindications to the use of near-infrared fluorescence imaging. In Part 1 of the study, Pegsitacianine is used to image primary tumors in patients with HNSCC to verify the diagnostic performance of Pegsitacianine fluorescence imaging for detecting primary tumors and metastatic lymph nodes. In Part 2, Pegsitacianine is used in patients with unknown primary cancer of the head and neck.

All participants received a single intravenous dose of Pegsitacianine (1 mg/kg) 6–300 hours prior to robotic surgery. Intraoperative fluorescence imaging was performed using FDA-approved and investigational near-infrared imaging systems. Additional imaging was conducted on resected tissues in the operating room using multiple camera platforms on the back table. Following image-guided resection, all specimens were submitted for standard pathological evaluation. In three participants, resected UPC tissues were further analyzed using the Xenium spatial transcriptomic platform (10x Genomics).

All participants provided written informed consent prior to enrollment. The study protocol was reviewed and approved by the Institutional Review Board of the University of Texas Southwestern Medical Center (IRB: STU-2022–0406, STU-092013–032).

### Three-dimensional imaging of ICG in human tumor

Patient tissue specimens (~1 mm^3^) were fixed in 4% paraformaldehyde (PFA) in phosphate-buffered saline (PBS) at 4 °C overnight and subsequently washed three times with PBS. Samples were permeabilized and blocked in PBS containing 5% bovine serum albumin (BSA) and 0.02% digitonin for 24 h at room temperature with gentle agitation to enhance antibody penetration. Fluorophore-conjugated primary antibodies against pan-cytokeratin (1:150) were diluted in the same blocking buffer and incubated with tissues for 48–72 hour at 37 °C with gentle rocking. Following staining, tissues were dehydrated through a graded ethanol series (50%, 70%, and 100%; 30 min each) and delipidated in xylene (2 × 1 h). Samples were then optically cleared in a benzyl alcohol/benzyl benzoate (BABB, 1:2 v/v) solution until fully transparent.

Three-dimensional imaging was performed using a custom-built light sheet fluorescence microscope equipped with a 785 nm diode laser. Whole-tissue scans were acquired with a 4× objective (NA 0.13) at a z-step of 1.6 μm to capture the complete tissue volume. Final 3D reconstruction and quantitative image analysis were conducted in Imaris 10.2 software (Oxford Instruments) using volume rendering and surface reconstruction modules. Multichannel datasets were visualized with distinct color mappings to facilitate spatial correlation analyses between fluorescence signals.

### Data acquisition for single cell spatial analysis of ICG signal and transcriptomics

Formalin-fixed, paraffin-embedded (FFPE) tissue specimens were obtained from Pegsitacianine-guided resections. For each case, three consecutive serial sections were collected to enable integrated analysis of indocyanine green (ICG) fluorescence and spatial transcriptomics. The central section (slide 2) was processed for spatial transcriptomic profiling using the 10x Genomics Xenium In Situ platform with the Human 5K Pan-Tissue & Pathways Panel, following the manufacturer’s protocol.

The adjacent sections (slides 1 and 3) were used to acquire ICG fluorescence signals. Briefly, slides were deparaffinized through a graded series of xylene, ethanol, and water washes, counterstained with DAPI (Spectral DAPI, Akoya), and mounted using ProLong^™^ Diamond Antifade Mountant (Invitrogen). After drying, fluorescence imaging was performed using the Akoya PhenoImager with excitation at the 780 nm channel.

### Integration of fluorescence imaging with spatial transcriptomics

Fluorescence images were aligned to the Xenium-analyzed tissue section using *Warpy*^34^. A composite acidity map was generated by averaging the ICG fluorescence signals from the flanking serial sections (slides 1 and 3) and applying a Gaussian smoothing filter (**Extended Data Fig. 4a**). Pixel-level fluorescence intensity was mapped to individual cells using rasterized segmentation boundaries derived from Xenium cell segmentation. The resulting mean fluorescence intensity (MFI) per cell was then incorporated into the corresponding AnnData object for downstream integrative spatial analysis.

### Analysis and plotting of integrated single-cell data set

Gene expression data from Xenium was analyzed using the Scanpy (v1.11.0)^35^ scRNA-seq analysis pipeline and visualized with Squidpy (v.1.6.5)^36^. Expression matrices were filtered to remove low-quality data, excluding cells with fewer than 40 total counts or 15 uniquely expressed genes, and genes expressed in fewer than 3 cells. Normalization, variable gene selection, and scaling were performed using Scanpy with default parameters, except for the normalization target sum, which was set to 10000. Cross-sample expression integration was performed using scvi-tools (v1.3.0)^37^ with default parameters. Neighborhood graph analysis was conducted with n_neighbors=50, and clustering was performed using the Leiden algorithm with Scanpy’s default parameters. Cell type annotation was guided by canonical marker gene expression and manually curated based on spatial context and expression patterns. Marker gene lists for each annotated cell type were generated using the rank_genes_groups function in Scanpy with default parameters.

Spatial enrichment of metabolic gene signatures, including glycolysis, the tricarboxylic acid (TCA) cycle, and acid transport pathways, was assessed using gene set scoring. Gene sets were selected based on their availability within the Xenium Prime 5K Pan-Tissue & Pathways Panel and cross-referenced with the MSigDB Gene Set Enrichment Analysis (GSEA). The complete list of genes used for pathway scoring is provided below.

Acidotic metabolism: HK2, ENO2, PGK1, PKM, ALDOA, HK1, PFKFB3, PDHA1, PDHB, DLD, CYCS, ATP5F1A, ATP5F1C, SDHA, SDHB, NDUFAB1, LDHA, SLC16A1, CA9, CA12.

### Statistical analysis

Differential gene expression analysis for cell type annotation was performed using Scanpy (v1.11.0). Sensitivity and specificity of Pegsitacianine fluorescence relative to histopathology were calculated from confusion matrices, and receiver operating characteristic (ROC) curves were generated using GraphPad Prism (v9.5.1). Cross-group comparisons were performed using one-way or two-way ANOVA as appropriate, also in GraphPad Prism (v9.5.1). A *P* value < 0.05 was considered statistically significant.

## Supplementary Material

Supplementary Files

This is a list of supplementary files associated with this preprint. Click to download.

- ExtendedDataFigureandtableS1.docx
- SupplementaryVideo1Patient6.mp4
- SupplementaryVideo2Patient8.mp4
- SupplementaryVideo3Patient5.mp4
- SupplementaryVidoe4Patient14.mp4
- SupplementaryVidoe5Patient15.mp4

## Data Availability

Processed spatial transcriptomic dataset has been deposited in Zenodo under accession number [17727830] [https://zenodo.org/records/17727830], with restricted access for reviewers and public release upon publication. The file can be downloaded through temporary link: [https://zenodo.org/records/17727830?token=eyJhbGciOiJIUzUxMiJ9.eyJpZCI6ImVmZDk1YjY0LWU5MzItNDRkNS04NGNiLWM3Mzg3YWViY2ZjMCIsImRhdGEiOnt9LCJyYW5kb20iOiJmNGZjZGQ4YmUyMTNiNGQ4ZDYyZGM0MDRhNmFmM2NmNSJ9.Fi5GQWw8s8dwvZcFmf11oPTDy1ZlQtJxcXG9gucwFClu4ifbZ9KzZEh0dOI6SQrjZ0cSdzrE WyGPGCwMuCAMlA] Additional patient-related data are available upon reasonable request to the corresponding authors and are subject to approval by the Institutional Review Board of UT Southwestern Medical Center.

## Code availability

The data analysis was performed using standard python function or Scanpy function without customized design. The integration of ICG data to single cell spatial data was performed using MASK2SC workflow^25^ and is available at https://github.com/ba-sketcvh/Mask2SC.

## References

[1] Johnson D.E. Head and neck squamous cell carcinoma. Nat. Rev. Dis. Primers 6, 92 (2020). 33243986 10.1038/s41572-020-00224-3 PMC7944998

[2] Ferris R.L. & Westra W. Oropharyngeal Carcinoma with a Special Focus on HPV-Related Squamous Cell Carcinoma. Annu. Rev. Pathol. 18, 515–535 (2023). 36693202 10.1146/annurev-pathmechdis-031521-041424 PMC11227657

[3] Greco F.A. Molecular profiling in unknown primary cancer: accuracy of tissue of origin prediction. Oncologist 15, 500–506 (2010). 20427384 10.1634/theoncologist.2009-0328 PMC3227979

[4] Ota I. & Kitahara T. Cancer of unknown primary in the head and neck: Diagnosis and treatment. Auris Nasus Larynx 48, 23–31 (2021). 32888761 10.1016/j.anl.2020.08.014

[5] Chin R.-I. Detection of solid tumor molecular residual disease (MRD) using circulating tumor DNA (ctDNA). Mol. Diagn. Ther. 23, 311–331 (2019). 30941670 10.1007/s40291-019-00390-5 PMC6561896

[6] Tivey A., Church M., Rothwell D., Dive C. & Cook N. Circulating tumour DNA—looking beyond the blood. Nature reviews clinical oncology 19, 600–612 (2022). 10.1038/s41571-022-00660-y PMC9341152 35915225

[7] Morris G.J. Cancer of unknown primary site. Semin. Oncol. 37, 71–79 (2010). 20494696 10.1053/j.seminoncol.2010.03.009

[8] Wilhelm S. Analysis of nanoparticle delivery to tumours. Nature Reviews Materials 1, 16014 (2016).

[9] Nguyen L.N.M. The mechanisms of nanoparticle delivery to solid tumours. Nature Reviews Bioengineering 2, 201–213 (2024). 10.1038/s44222-024-00203-3 PMC7616668 39376248

[10] He H., Liu L., Morin E.E., Liu M. & Schwendeman A. Survey of Clinical Translation of Cancer Nanomedicines—Lessons Learned from Successes and Failures. Acc. Chem. Res. 52, 2445–2461 (2019). 31424909 10.1021/acs.accounts.9b00228

[11] Rayamajhi S. pH-responsive cationic liposome for endosomal escape mediated drug delivery. Colloids Surf. B. Biointerfaces 188, 110804 (2020). 31972443 10.1016/j.colsurfb.2020.110804

[12] Hatakeyama H., Akita H. & Harashima H. A multifunctional envelope type nano device (MEND) for gene delivery to tumours based on the EPR effect: A strategy for overcoming the PEG dilemma. Adv. Drug Delivery Rev. 63, 152–160 (2011). 10.1016/j.addr.2010.09.001 20840859

[13] Matsumura Y., Oda T. & Maeda H. General mechanism of intratumor accumulation of macromolecules: advantage of macromolecular therapeutics. Gan to kagaku ryoho. Cancer & chemotherapy 14, 821–829 (1987). 2952066

[14] Nguyen L.N.M. The exit of nanoparticles from solid tumours. Nat. Mater. 22, 1261–1272 (2023). 37592029 10.1038/s41563-023-01630-0

[15] Tylawsky D.E. P-selectin-targeted nanocarriers induce active crossing of the blood–brain barrier via caveolin-1-dependent transcytosis. Nat. Mater. 22, 391–399 (2023). 36864161 10.1038/s41563-023-01481-9 PMC9981459

[16] Faubert B., Solmonson A. & DeBerardinis R.J. Metabolic reprogramming and cancer progression. Science 368, 152 (2020). 10.1126/science.aaw5473 PMC7227780 32273439

[17] Hanahan D. & Weinberg R.A. Hallmarks of cancer: the next generation. Cell 144, 646–674 (2011). 21376230 10.1016/j.cell.2011.02.013

[18] Warburg O. The Metabolism of Carcinoma Cells1. The Journal of Cancer Research 9, 148–163 (1925).

[19] Gillies R.J., Raghunand N., Karczmar G.S. & Bhujwalla Z.M. MRI of the tumor microenvironment. J. Magn. Reson. Imaging 16, 430–450 (2002). 12353258 10.1002/jmri.10181

[20] Savic L.J. Molecular Imaging of Extracellular Tumor pH to Reveal Effects of Locoregional Therapy on Liver Cancer Microenvironment. Clin Cancer Res 26, 428–438 (2020). 31582517 10.1158/1078-0432.CCR-19-1702 PMC7244230

[21] Volk T., Jahde E., Fortmeyer H.P., Glusenkamp K.H. & Rajewsky M.F. Ph in Human Tumor Xenografts - Effect of Intravenous Administration of Glucose. Br. J. Cancer 68, 492–500 (1993). 8353039 10.1038/bjc.1993.375 PMC1968383

[22] Feng Q. Severely polarized extracellular acidity around tumour cells. Nat Biomed Eng 8, 787–799 (2024). 38438799 10.1038/s41551-024-01178-7 PMC12192457

[23] Voskuil F.J. Exploiting metabolic acidosis in solid cancers using a tumor-agnostic pH-activatable nanoprobe for fluorescence-guided surgery. Nat. Commun. 11, 3257 (2020). 32591522 10.1038/s41467-020-16814-4 PMC7320194

[24] Huang G. PET imaging of occult tumours by temporal integration of tumour-acidosis signals from pH-sensitive (64)Cu-labelled polymers. Nat. Biomed. Eng. 4, 314–324 (2020). 31235828 10.1038/s41551-019-0416-1 PMC6928453

[25] Chen J. Protocol for integrating immunohistochemistry and H&E annotations with Xenium data at single-cell resolution. STAR Protocols 6, 104107 (2025). 41014563 10.1016/j.xpro.2025.104107 PMC12509896

[26] Lin Z.P. Macrophages Actively Transport Nanoparticles in Tumors After Extravasation. ACS Nano 16, 6080–6092 (2022). 35412309 10.1021/acsnano.1c11578

[27] Machtay M. Factors Associated With Severe Late Toxicity After Concurrent Chemoradiation for Locally Advanced Head and Neck Cancer: An RTOG Analysis. J. Clin. Oncol. 26, 3582–3589 (2008). 18559875 10.1200/JCO.2007.14.8841 PMC4911537

[28] Orland M.D., Ullah F., Yilmaz E. & Geiger J.L. Immunotherapy for Head and Neck Squamous Cell Carcinoma: Present and Future Approaches and Challenges. JCO Oncology Practice 20, 1588–1595 (2024). 38709998 10.1200/OP.24.00041

[29] Peer D. Nanocarriers as an emerging platform for cancer therapy. Nat. Nanotechnol. 2, 751–760 (2007). 18654426 10.1038/nnano.2007.387

[30] Gabizon A.A., Gabizon-Peretz S., Modaresahmadi S. & La-Beck N.M. Thirty years from FDA approval of pegylated liposomal doxorubicin (Doxil/Caelyx): an updated analysis and future perspective. BMJ Oncol 4, e000573 (2025). 10.1136/bmjonc-2024-000573 PMC11751825 39885941

[31] Chauhan V.P. & Jain R.K. Strategies for advancing cancer nanomedicine. Nat. Mater. 12, 958–962 (2013). 24150413 10.1038/nmat3792 PMC4120281

[32] Lammers T., Kiessling F., Hennink W.E. & Storm G. Drug targeting to tumors: Principles, pitfalls and (pre-) clinical progress. J. Controlled Release 161, 175–187 (2012). 10.1016/j.jconrel.2011.09.063 21945285

[33] Sindhwani S. The entry of nanoparticles into solid tumours. Nat. Mater. 19, 566–575 (2020). 31932672 10.1038/s41563-019-0566-2

[34] Chiaruttini N. An Open-Source Whole Slide Image Registration Workflow at Cellular Precision Using Fiji, QuPath and Elastix. Frontiers in Computer Science 3 (2022).

[35] Wolf F.A., Angerer P. & Theis F.J. SCANPY: large-scale single-cell gene expression data analysis. Genome Biol. 19, 15 (2018). 29409532 10.1186/s13059-017-1382-0 PMC5802054

[36] Palla G. Squidpy: a scalable framework for spatial omics analysis. Nat. Methods 19, 171–178 (2022). 35102346 10.1038/s41592-021-01358-2 PMC8828470

[37] Gayoso A. A Python library for probabilistic analysis of single-cell omics data. Nat. Biotechnol. 40, 163–166 (2022). 35132262 10.1038/s41587-021-01206-w

